# Establishment of defined daily doses for veterinary antimicrobials in food-producing animals in the Republic of Korea and comparison with European Medicines Agency values

**DOI:** 10.1099/jmm.0.002209

**Published:** 2026-10-05

**Authors:** Seunghoe Kim, Ju-yeon You, Hyejeong Kang, Suk-Kyung Lim, Yun Sang Cho, Hyun-Mi Kang

**Affiliations:** 1 Bacterial Diseases Division, Animal and Plant Quarantine Agency, 177 Hyeoksin 8-ro, Gimcheon-si, Gyeongsangbuk-do 39660, Republic of Korea

**Keywords:** antimicrobial use (AMU), defined daily doses, defined daily doses for animals (DDDvet), defined daily doses for Republic of Korea (DDDkr), quotient of daily defined doses (QDDD)

## Abstract

**Introduction.** Antimicrobials are essential for maintaining animal health; however, their use exerts selective pressure that promotes antimicrobial resistance. Therefore, monitoring antimicrobial use (AMU) is critical for managing the emergence and selection of resistance and various metrics have been developed in veterinary medicine to monitor these usage patterns.

**Gap statement.** Although metrics such as sales volume are widely used, they do not account for differences in antimicrobial potency. Currently, no defined daily dose metric specific to food-producing animals in Korea has been established to accurately assess domestic usage patterns.

**Aim.** In this study, we aimed to establish defined daily doses for Republic of Korea (DDDkr) for antimicrobial agents approved for use in cattle, pigs and poultry and to compare these values with the defined daily doses for animals (DDDvet) established by the European Medicines Agency (EMA).

**Methodology.** A total of 931 veterinary antimicrobial products approved and marketed with recorded sales volumes for food-producing animals in the Republic of Korea as of 2023 were analysed. DDDkr were defined following EMA principles, based on species, administration route and antimicrobial agents. To quantitatively compare the derived DDDkr values with existing DDDvet standards, the quotient of daily defined doses (QDDD) was calculated.

**Results.** We established 200 DDDkr values: 80 for cattle, 80 for pigs and 40 for poultry. Existing DDDvet values covered 57.5% of Korean veterinary antimicrobials, as certain formulations (sprays, intramammary and intrauterine products) lacked comparable EMA values. Median QDDD values were 0.87 for cattle, 0.79 for pigs and 0.75 for poultry, reflecting significant differences between DDDkr and DDDvet for cattle and pigs (*P*<0.05), whereas no significant difference was observed in poultry. Comparative analysis showed that most QDDD values were less than 1.

**Conclusion.** This study is the first to establish a DDDkr for food-producing animals in Korea. The results indicate that national dosages are generally lower than those defined in European standards, highlighting the need for country-specific metrics such as DDDkr to ensure more accurate domestic AMU monitoring.

## Introduction

 Antimicrobials are essential medicinal products used in food-producing animals to ensure animal health and welfare by treating, controlling and preventing infectious diseases [1, 2]. However, widespread administration of these agents exerts selective pressure that favours the emergence and spread of antimicrobial-resistant bacteria, which poses a significant threat to global public health [3, 4]. Recognizing this risk, international organizations and national governments have prioritized the optimization and reduction of antimicrobial use (AMU) [2, 5–11]. Accurate measurement and continuous monitoring of AMU are essential for the design, implementation and evaluation of effective antimicrobial stewardship strategies and resistance mitigation policies [6, 12]. To achieve this, establishing robust surveillance systems based on standardized and comparable quantitative metrics is crucial. Consequently, various metrics and data collection methods are currently employed by different national and international surveillance systems to monitor AMU trends [12–15].

Among the diverse AMU indicators used globally, weight- and dose-based metrics are the most prominent [5, 6, 12]. Weight-based indicators include the total mass of active substances, milligram per population correction unit employed by the European Medicines Agency (EMA) [6], and milligram per kilogram of animal biomass adopted by the World Organisation for Animal Health [5]. In contrast, dose-based metrics include the prescribed daily doses and the used daily doses, which measure the intended prescription and the actual amount administered at the farm level, respectively [6, 15, 16]. However, these metrics present distinct limitations for national-level surveillance. Although weight-based metrics facilitate broad international comparisons, they do not account for variations in antimicrobial potency and formulation, thereby failing to capture the true intensity of therapeutic exposure [6, 13, 16, 17]. Conversely, although dose-based metrics offer the highest accuracy, the labour-intensive and complex nature of collecting granular prescriptions and use data makes their application significantly challenging on a national scale [6, 16].

To bridge the gap between practical data collection and precise usage estimation, the EMA adopted the concept of the defined daily doses (DDD) from human medicine for veterinary practice, formally establishing defined daily doses for animals (DDDvet) in 2016 [18–20]. DDDvet is defined as the assumed average maintenance dose per kilogram of animal body weight per day for a specific species and serves as a crucial standardized technical unit [18, 20]. Unlike weight-based metrics, DDDvet inherently corrects for variations in pharmacological potency and administration routes, allowing for a more accurate reflection of therapeutic intensity [18, 20]. However, due to discrepancies in recommended doses specified in product-specific summaries of product characteristics, establishing standardized daily doses at the active-substance level requires critical compromises, such as adopting a median, mean or maximum value [21]. Consequently, discrepancies between these standardized values and actual doses used can lead to either an underestimation or overestimation of total antimicrobial consumption, presenting a notable drawback of this approach [21]. Furthermore, it enables the estimation of population-level antimicrobial exposure using readily accessible aggregated national sales data, eliminating the need for labour-intensive farm-level records [18, 22].

Currently, South Korea is reporting weight-based sales data; however, no DDD metric specific to domestic food-producing animals has been established. In this study, we aimed to develop defined daily doses for the Republic of Korea (DDDkr) for approved and marketed single or combination veterinary antimicrobial active substances, serving as a standardized metric for national surveillance, and to compare these values with the EMA’s DDDvet values.

## Methods

### Defined daily doses in Korea

DDDkr values were established at the active substance level following the principles of the EMA, with modifications to align with the domestic context [18]. As of 2023, data on veterinary antimicrobial products approved and commercially available for use in cattle, pigs and poultry in the Republic of Korea was obtained from the Korea Animal Health Products Association. For each product, information regarding the recommended daily dosage, recommended treatment duration and approved indications was collected directly from the product labels.

The baseline calculations for these values were determined by the target species, route of administration and whether the formulation was a single-ingredient or combination-ingredient product. If two or more commercially available products contained the same active substance, the final DDD value was calculated as the arithmetic mean of their recommended daily dosages. For products with varying dosages across different indications, the arithmetic mean of the indication-specific doses was applied. In cases where the dosage varied over the treatment period (such as a different dose for the first few days), a time-weighted average was calculated across the entire recommended treatment duration. For combination-ingredient antimicrobials, DDD values were assigned separately to each active substance according to the EMA guidelines, considering the synergy of antimicrobial combinations.

Specific calculation methodologies were applied based on the administration route. For formulations administered through feed or water, dosages were converted to milligram per kilogram of body weight using standard conversion rates published by the EMA [18]. For intramammary and intrauterine products, daily doses in mg kg^−1^ day^−1^ were calculated by dividing the total antimicrobial dose by a standard cattle body weight of 635 kg, in accordance with the Japanese methodology [22], and further adjusting this value by the application period (either an assumed 4 days or the specific duration recommended on the product label). For spray formulations, DDDkr values were determined based on the daily dosage (mg kg^−1^ body weight day^−1^) specified on the approved product labels in the Republic of Korea.

### Quotient of Korean and European values (QDDD)

The quotient of daily defined doses (QDDD) was calculated by comparing the DDD values in Korea and Europe [20] as follows:


$$QDDD=\frac{DDDkr}{DDDvet}$$

A QDDD of 1 indicates identical dosages between the two systems, whereas values greater or less than 1 indicate higher or lower Korean dosages, respectively. A comparative analysis was performed only when the corresponding DDDvet values were available. The EMA values for the antimicrobials were excluded from comparison when unavailable. Intramammary and intrauterine products were not compared because DDDvet was not available in mg kg^−1^ day^−1^.

### Statistical analysis

Statistical analysis was performed to evaluate differences between the established DDDkr and European DDDvet values using R Statistical Software (version 4.4.2, R Foundation for Statistical Computing, Vienna, Austria). The Wilcoxon signed-rank test was used for paired samples to determine the statistical significance of the differences between the two metrics. A *P*-value≤0.05 was considered statistically significant.

## Results

### Analysis of veterinary antimicrobials for food-producing animals in Korea

A total of 931 antimicrobial products comprising 53 active substances commercially available for food-producing animals in the Republic of Korea as of 2023 were analysed (Table 1, Data S1–S3, available in the online Supplementary Material). Most of these products were approved for pigs (*n*=842), followed by cattle (*n*=431) and poultry (*n*=384). Oral formulations were predominant, accounting for 68.6% (*n*=639) of all products, whereas injectable formulations accounted for 27.8% (*n*=259). Other formulations included intramammary (*n*=22), topical (*n*=9) and intrauterine (*n*=2) products (Table 1). Florfenicol was the most frequently approved active substance (*n*=133), followed by amoxicillin (*n*=85) and tiamulin (*n*=75) (Data S1–S3). Based on these product profiles, 200 DDDkr values were established, including 103 values for oral administration, 72 for injection, 18 for intramammary administration, 5 for topical administration and 2 for intrauterine administration (Table 2, Data S1–S3).

**Table 1.** Number of antimicrobial products for cattle, pigs and poultry in Korea by administration route and number of active substances contained

Animal	Product type	Administration route					Total
		Oral	Injection	Intramammary	Intrauterine	Spray	
Cattle	Single	150	193	11	2	0	356
	Combination	26	36	11	0	2	75
	Sub-total (%)	176 (40.8)	229 (53.1)	22 (5.1)	2 (0.5)	2 (0.5)	431 (100)
Pig	Single	462	202	0	0	7	671
	Combination	129	40	0	0	2	171
	Sub-total (%)	591 (70.2)	242 (28.7)	0 (0)	0 (0)	9 (1.1)	842 (100)
Poultry	Single	295	9	0	0	0	304
	Combination	77	3	0	0	0	80
	Sub-total (%)	372 (96.9)	12 (3.1)	0 (0)	0 (0)	0 (0)	384 (100)
Total	Single	500	213	11	2	7	733
	Combination	139	46	11	0	2	198
	Total (%)	639 (68.6)	259 (27.8)	22 (2.4)	2 (0.2)	9 (1.0)	931 (100)

**Table 2.** Number of established DDDkr values for cattle, pigs and poultry in Korea by administration route and number of active substances contained

Product type	Animal	Administration route					Total
		Oral	Injection	Intramammary	Intrauterine	Spray	
Single	Cattle	8	17	7	2	0	34
	Pig	22	17	0	0	1	40
	Poultry	18	3	0	0	0	21
	Sub-total (%)	48 (50.5)	37 (38.9)	7 (7.4)	2 (2.1)	1 (1.1)	95 (100)
Combination	Cattle	15	18	11	0	2	46
	Pig	23	15	0	0	2	40
	Poultry	17	2	0	0	0	19
	Sub-total (%)	55 (52.4)	35 (33.3)	11 (10.5)	0 (0)	4 (3.8)	105 (100)
	Sub-total (%)	103 (51.5)	72 (36)	18 (9)	2 (1)	5 (2.5)	200 (100)

### Comparison of DDDkr with European DDDvet for antimicrobial agents in cattle

A total of 80 DDDkr values were defined for 45 antimicrobial agents for cattle (Table 2, Data S1). The route of administration with the most assigned DDDkr was injection, which was defined in 35 cases. This was followed by oral, intramammary, intrauterine and topical administration. Examination of the daily dose distribution revealed a wide range of doses (Table 3, Data S1). The highest-selling antimicrobial agent [23], amoxicillin, was defined as 7.1 mg kg^−1^ in single oral administration, 5.7 mg kg^−1^ in combination oral administration and 13.1 mg kg^−1^ in single injection administration. The antimicrobial agent with the largest inter-product variation in recommended doses was spiramycin, which ranged from 3 to 50 mg kg^−1^ and differed by 1,667% (Data S1). Sixty-nine percent of DDDkr values for cattle had the same mean and median values (Data S1). A detailed list of DDDkr values is provided in Data S1.

**Table 3.** Comparison of DDDkr and DDDvet values for major antimicrobial agents used in cattle

Class	Antimicrobial agent	Administration route	Product type	Count	DDDkr	Median	Minimum	Maximum	DDDkr	DDDvet	QDDD
Aminoglycosides	Neomycin	Oral	Single	2	8.9	8.9	7.7	10.0	17.7	21	0.42
			Combination	3	2.4	2.6	2.0	2.6	8.4	–	–
		Injection	Combination	1	7.5	7.5	7.5	7.5	7.5	7.4	1.01
		Intramammary	Combination	8	0.8	0.6	0.4	1.9	2.1	–	–
Amphenicols	Florfenicol	Injection	Single	16	18.8	20.0	10.0	20.0	42.2	13	1.44
Cephalosporins	Ceftiofur	Injection	Single	40	1.0	1.0	1.0	1.0	3.0	1	1.00
		Intramammary	Combination	1	0.6	0.6	0.6	0.6	2.5	–	–
Macrolides	Tylosin	Oral	Single	18	0.2	0.2	0.2	0.2	4.2	41	0.005
		Injection	Single	5	8.6	7.0	7.0	15.0	22.4	13	0.66
			Combination	3	4.9	5.0	2.6	7.0	11.3	–	–
Penicillins	Amoxicillin	Oral	Single	48	7.1	7.5	6.5	9.8	31.2	20	0.35
			Combination	3	5.7	3.3	3.3	10.5	22.7	–	–
		Injection	Single	15	13.1	15.0	7.0	15.0	23.6	8.3	1.58
	Ampicillin	Oral	Single	44	9.5	9.5	9.5	9.5	47.3	29	0.33
			Combination	14	5.0	5.0	5.0	5.0	25.0	–	–
		Injection	Single	11	7.8	7.5	7.5	11.3	10.3	11	0.71
			Combination	1	10.0	10.0	10.0	10.0	10.0	–	–
		Intramammary	Combination	1	0.4	0.4	0.4	0.4	1.6	–	–
	Penicillin	Injection	Single	4	5.2	4.0	3.6	9.0	11.6	14	0.37
			Combination	19	5.8	5.6	1.8	13.5	16.7	2.9	1.99
		Intramammary	Combination	1	0.4	0.4	0.4	0.4	0.4	–	–
Polypeptides	Colistin	Oral	Single	27	0.5	0.5	0.5	0.5	0.5	4.8	0.09
			Combination	17	0.3	0.2	0.2	0.6	1.5	–	–
		Injection	Combination	3	1.4	1.2	1.2	1.8	2.4	2.6	0.55
Quinolones	Enrofloxacin	Oral	Single	1	3.8	3.8	3.8	3.8	15.0	4.7	0.80
		Injection	Single	31	4.5	4.2	3.8	7.5	16.5	4.2	1.07
		Intramammary	Single	1	2.5	2.5	2.5	2.5	7.6	–	–
Tetracyclines	Oxytetracycline	Oral	Single	9	22.0	22.0	21.6	22.2	230.8	20	1.10
			Combination	2	26.0	26.0	26.0	26.0	104.0	–	–
		Injection	Single	5	15.5	20.0	8.8	20.0	15.5	6.5	2.39

The comparison between DDDkr and DDDvet in cattle involved the analysis of 41 pairs (51%) of oral and injectable antimicrobial values. Intramammary and intrauterine antimicrobials were excluded from the comparison because of differences in calculation methods. More than half of the DDDkr values (61%) were lower than the DDDvet values, whereas only 32% were higher. Notably, a difference of over 50% was observed in 15 comparison values (37%) (Fig. 1a and S1). Among the commonly used antimicrobial agents for Korean cattle, the DDDkr for oral amoxicillin (QDDD=0.35), oral ampicillin (QDDD=0.33), injectable ampicillin (QDDD=0.71), tylosin (QDDD=0.005) and injectable tylosin (QDDD=0.66) were all lower than the EMA values. However, the DDDkr value for injectable amoxicillin (QDDD=1.58) was higher than that for DDDvet (Table 3). The DDDkr values for cattle differed significantly from the DDDvet values (*P*=0.0388), with a median QDDD of 0.87.

**Fig. 1.**
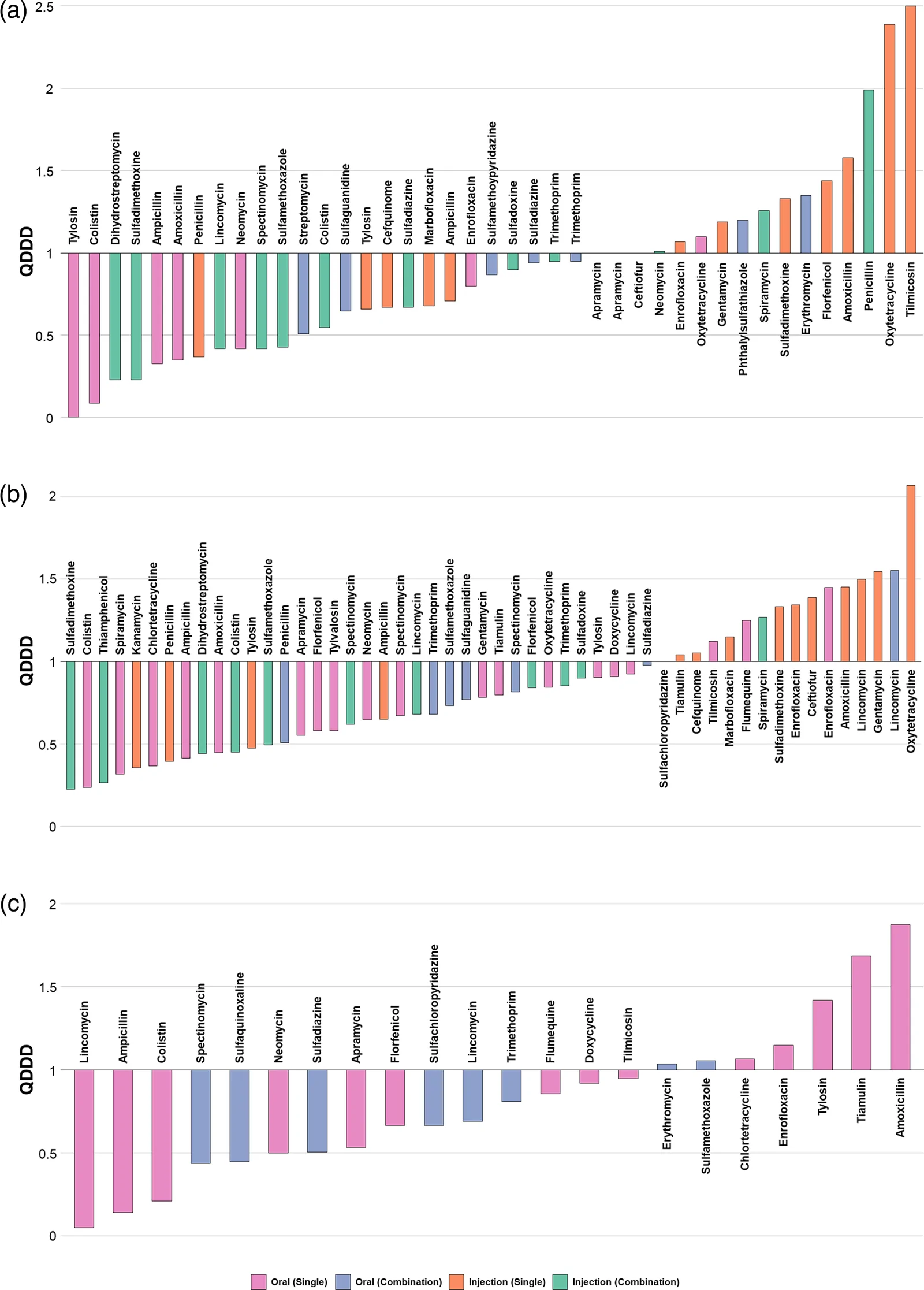
Comparison of the DDDkr of antimicrobial agents approved for use in food-producing animals with the corresponding values of the EMA (DDDvet). (**a**) Cattle; (**b**) pigs; and (**c**) poultry.

### Comparison of DDDkr with European DDDvet for antimicrobial agents in pigs

A total of 80 DDDkr values were defined for 43 antimicrobial agents for pigs (Table 2, Data S2). The most common DDDkr route of administration was oral, with 45 defined values, followed by injectable administration and topical formulations. The investigation of the distribution of daily dosages revealed a wide range of dosages (Table 4). The highest-selling antimicrobial agent [23], florfenicol, was defined as 5.8 mg kg^−1^ in single oral administration, 14.1 mg kg^−1^ in single injection administration and 8 mg kg^−1^ in combination injection administration. The antimicrobial with the largest variation in recommended dosages among the products was oral florfenicol, with a range of 1.2–10 mg kg^−1^, differing by 833% (Table 3, Data S2). Forty-nine percent of DDDkr values for pigs had the same mean and median values (Data S2). A detailed list of DDDkr values was provided in Data S2.

**Table 4.** Comparison of DDDkr and DDDvet values for major antimicrobial agents used in pigs

Class	Antimicrobial agent	Administration route	Product type	Count	DDDkr	Median	Minimum	Maximum	DDDkr	DDDvet	QDDD
Amphenicols	Florfenicol	Oral	Single	107	5.8	10.0	1.2	10.0	34.7	10	0.58
		Injection	Single	16	14.1	15.0	7.5	15.0	31.6	–	–
			Combination	5	8.0	10.0	5.0	10.0	19.2	9.5	0.84
Macrolides	Tilmicosin	Oral	Single	54	16.9	20.0	10.0	20.0	184.1	15	1.12
	Tylosin	Oral	Single	32	10.8	4.4	4.4	25.0	172.9	12	0.90
			Combination	13	4.4	4.4	4.4	4.4	92.4	–	–
		Injection	Single	5	6.2	6.0	6.0	7.0	16.1	13	0.48
			Combination	10	3.4	2.5	2.2	6.0	8.1	–	–
Penicillins	Amoxicillin	Oral	Single	64	7.6	7.5	6.5	20.0	34.6	17	0.45
			Combination	4	5.1	3.3	3.3	10.5	20.3	–	–
		Injection	Single	16	12.9	15.0	7.0	15.0	24.3	8.9	1.45
	Ampicillin	Oral	Single	44	12.5	12.5	12.5	12.5	62.2	30	0.42
			Combination	14	5.0	5.0	5.0	5.0	25.0	–	–
		Injection	Single	11	7.8	7.5	7.5	11.3	10.3	12	0.65
			Combination	1	10.0	10.0	10.0	10.0	10.0	–	–
Polypeptides	Bacitracin	Oral	Single	17	7.2	4.0	4.0	14.0	18.2	–	–
Pleuromutilins	Tiamulin	Oral	Single	49	7.7	6.2	3.2	12.2	62.1	9.7	0.80
			Combination	24	3.2	3.3	0.9	4.0	19.9	–	–
		Injection	Single	2	12.5	12.5	12.5	12.5	40.6	12	1.04
Sulfanamides	Sulfamethazine	Oral	Combination	18	4.9	4.4	4.4	8.8	90.7	–	–
	Sulfamethoxazole	Oral	Combination	29	14.7	18.0	4.0	26.7	68.0	20	0.74
		Injection	Combination	2	9.9	9.9	6.5	13.3	29.8	20	0.50
Tetracyclines	Chlortetracycline	Oral	Single	13	11.4	8.8	6.0	22.0	115.9	31	0.37
			Combination	24	9.2	9.9	4.0	10.0	62.4	–	–

The comparison of DDDkr and DDDvet in pigs involved the evaluation of 52 pairs (65%) of oral and injectable antimicrobials. More than half the DDDkr values (69%) were lower than the DDDvet values, whereas 29% were higher. Notably, a difference of more than 50% was observed in 16 comparison values (31%) (Figs 1b and S2). Among the commonly used antimicrobials in Korean pigs, the DDDkr for oral amoxicillin (QDDD=0.45), oral florfenicol (QDDD=0.58), injectable florfenicol (QDDD=0.84), oral tylosin (QDDD=0.9) and injectable tylosin (QDDD=0.48) were lower than the EMA values. In contrast, the daily dose of injectable amoxicillin (QDDD=1.45) was higher than the EMA values (Table 4). A significant difference was observed between DDDkr and DDDvet in pigs (*P*=0.000678), with a median QDDD of 0.79.

### Comparison of DDDkr with European DDDvet for antimicrobial agents in poultry

A total of 40 DDDkr values were defined for 29 antimicrobial agents for poultry (Table 2, Data S3). The commonly assigned DDDkr route of administration was oral, with 35 defined values, followed by injectable administration. An investigation of the distribution of daily dosages revealed a wide range of dosages (Table 5). Florfenicol, the highest-selling antimicrobial agent [23], was defined as 20 mg kg^−1^ in single oral administration. Oral spectinomycin exhibited the largest variation in recommended dosages among products, with a range of 8.6–76.8 mg kg^−1^ and differing by 895% (Data S3). Sixty-three percent of DDDkr values for poultry had the same mean and median values. A detailed list of DDDkr values was provided in Data S3.

**Table 5.** Comparison of DDDkr and DDDvet values for major antimicrobial agents used in poultry

Class	Antimicrobial agent	Administration route	Product type	Count	DDDkr	Median	Minimum	Maximum	DDDkr	DDDvet	QDDD
Amphenicol	Florfenicol	Oral	Single	56	20.0	20.0	20.0	20.0	100.7	30	0.67
Lincosamide	Lincomycin	Oral	Single	9	0.4	0.4	0.4	0.4	0.4	8.6	0.05
			Combination	23	15.2	11.4	8.6	38.3	241.3	22	0.69
		Injection	Combination	3	21.7	10.0	10.0	45.0	65.0	–	–
Macrolide	Tiamulin	Oral	Single	26	38.8	43.1	10.4	43.1	161.2	23	1.69
			Combination	9	9.3	8.6	8.6	15.2	51.7	–	–
		Injection	Single	1	2.5	2.5	2.5	2.5	2.5	–	–
	Tilmicosin	Oral	Single	34	17.0	17.3	15.5	17.3	68.2	18	0.95
Penicillin	Amoxicillin	Oral	Single	26	30.0	30.0	15.0	45.0	141.9	16	1.88
			Combination	1	33.0	33.0	33.0	33.0	132.0	–	–
	Ampicillin	Oral	Single	44	15.1	15.0	15.0	20.0	75.2	108	0.14
			Combination	14	11.5	11.5	11.5	11.5	57.5	–	–
Polypeptide	Bacitracin	Oral	Single	20	8.6	6.3	3.9	21.5	25.7	–	–
	Colistin	Oral	Single	17	1.1	0.7	0.7	2.4	2.1	5.1	0.21
			Combination	20	3.1	1.6	1.4	9.0	15.2	–	–
Sulfanamide	Sulfamethoxazole	Oral	Combination	16	28.5	26.0	15.5	46.0	146.0	27	1.06
Tetracycline	Chlortetracycline	Oral	Single	14	32.0	33.6	13.2	50.1	208.0	30	1.07
			Combination	10	25.9	25.9	25.7	26.0	134.5	–	–

The comparison of DDDkr and DDDvet in poultry involved the evaluation of 22 pairs (55%) of oral antimicrobials. More than half of the DDDkr values (68%) were lower than the DDDvet values, whereas only 32% were higher. Notably, a difference of more than 50% was observed in seven comparison values (32%) (Figs 1c and S3). Among the commonly used antimicrobials for Korean poultry, the DDDkr values for florfenicol (QDDD=0.67) and ampicillin (QDDD=0.14) were lower than the EMA values for daily dosages. In contrast, the DDDkr for chlortetracycline (QDDD=1.07) was used at a similar daily dosage (Table 5). For poultry, the difference between DDDkr and DDDvet showed a numerical trend but was not statistically significant (*P*=0.0715), with a median QDDD of 0.75.

## Discussion

As the use of antimicrobials exerts selective pressure that favours the emergence of resistance, various standardized metrics and data collection methods are currently used by national and international surveillance systems to monitor usage trends and mitigate these risks [1–15]. In this study, DDDkr standards were developed for cattle, pigs and poultry for the first time for approved products containing complex antimicrobial active ingredients used in Korea, and DDDkr was compared with the DDDvet of the EMA.

The number of established DDDkr values for pigs and cattle in our study was 80 each, and the number of approved products for pigs was significantly higher than that for cattle. In addition, Japan had a similar pattern, where the number of antimicrobial products used for pigs was higher than that for cattle [22]. These numerical differences may be attributable to the high redundancy of products containing the same active ingredients within the pig industry because the market structure relies heavily on the availability of generic off-patent antimicrobials rather than the introduction of new treatment options [5, 6, 22]. In this study, we found that the ratio of administration routes differed depending on the livestock species. Pigs and poultry had high oral administration rates, whereas cattle had a high injection rate. Consistent with our findings, global trends indicate that oral products are predominantly used in pigs and poultry, whereas injectable products are prevalent in cattle [16, 22, 24, 25]. The differences in these species-specific administration routes are attributable to the unique management systems and physiological characteristics of each species [26, 27]. In the pig and poultry sectors, oral group drug treatment is preferred because of labour efficiency and the mass-rearing environment [26, 27]. Conversely, the high dependence on the injection route for cattle may be due to the feasibility of individual animal management and the clinical need to avoid disturbances in rumen microbial populations that may arise from oral administration [27].

In the present study, the DDDkr values were generally lower than those of the European EMA references (DDDvet) across all three animal species, with median QDDD values of 0.87 for cattle, 0.79 for pigs and 0.75 for poultry. This consistent trend of lower national dosages aligned with findings from other non-European countries, such as Japan (median QDDD: cattle 0.66, pigs 0.61) and Canada [16, 22]. In contrast, a study in European context like Switzerland reported QDDD values closer to 1.0 (median 0.91), indicating relatively minor deviations from EMA standards [28]. Further specific QDDD analysis revealed that 75% of the oral antimicrobials and 54% of the injectable antimicrobials had values less than 1. Low QDDD values were observed for oral tylosin in cattle, injectable sulfadimethoxine in pigs, and oral lincomycin in poultry. Conversely, exceptionally high QDDD values were observed for injectable tilmicosin in cattle, injectable oxytetracycline in pigs, and oral amoxicillin in poultry. Similar significant differences for certain agents have been reported in other countries, including Japan [22]. The difference between DDDvet and DDDkr may be attributable to several factors. First, these differences may arise due to variations in labelling regulations, treatment indications and husbandry practices across countries [16, 22]. For instance, regarding labelling regulations and husbandry practices, European product labels generally authorize individual medication via milk for calves, whereas Korean labels frequently approve group medication via feed, which may lead to a lower authorized dosage per kilogram of body weight [29, 30]. Second, the EMA’s DDDvet was derived from the average of the assigned dosages across nine EU member states. As authorized dosages for the same antimicrobial agent can vary significantly among nations, the inclusion of countries with higher approved dosages can distort upward the overall average, resulting in a higher baseline than the national criteria of a single country, such as Korea [18, 20]. For example, a previous European multi-country study documented that national product authorizations for swine antimicrobials vary among countries, with Germany approving significantly higher authorized daily dosages for key agents – such as amoxicillin, chlortetracycline and tiamulin – than nations like France, Belgium and Sweden [31]. Finally, the observed discrepancies could be attributed to outdated dosages [22]. In Australia, outdated antimicrobial labelling in horses and cattle was reported to lead to underdosing [32, 33]. In Japan, it was suggested that manufacturers preferred registering products with previously approved dosages rather than new ones to reduce time and cost burdens, hindering label updates to clinically effective dosages [22]. Similarly in Korea, seeking product approvals based on existing dosages rather than introducing revised dosing regimens remained a standard commercial practice, thereby perpetuating the use of historical label values. Determining all the factors that account for these discrepancies between DDDkr and DDDvet, however, was outside the boundaries of this study. Given that underdosing could accelerate the selection of resistant pathogens, these older products approved at very low doses might warrant systematic re-evaluation to align with modern antimicrobial stewardship guidelines. Given these fundamental discrepancies, the establishment of DDDkr provides a more appropriate baseline for national surveillance than DDDvet by capturing the current status of veterinary AMU in Korea more accurately. Country-specific metrics like DDDkr, however, may face limitations in direct cross-country evaluations due to methodological differences and product variations. These constraints simply highlight the complementary need for standardized global DDD values to facilitate broader international comparisons [16, 22].

In conclusion, DDDkr values were established by analysing veterinary antimicrobial products approved for use in cattle, pigs and poultry in Korea. The current DDDvet values do not cover all veterinary antimicrobials used in Korea, and a comparison of the defined DDDkr with the European DDDvet showed overall low QDDD values. The absence of DDDvet values for certain antimicrobial agents, combined with these results, suggests that DDDkr is a more suitable metric for accurately reflecting AMU in the Korean livestock industry. Given the methodological differences and differences in approved antimicrobial agents, it is necessary for each country to develop country-specific metrics, such as DDDkr, for effective and precise national AMU monitoring.

## Supplementary material

10.1099/jmm.0.002209 Supplementary Material 1.

## Abbreviations

AMU: antimicrobial use
DDD: defined daily dose
DDDkr: defined daily doses for the Republic of Korea
DDDvet: defined daily doses for animals
EMA: European Medicines Agency
QDDD: quotient of daily defined doses
