# Supplementary material for: Establishment of defined daily doses for veterinary antimicrobials in food-producing animals in the Republic of Korea and comparison with European Medicines Agency values
Source: J Med Microbiol. 2026 Oct 5;75(10):002209. doi: 10.1099/jmm.0.002209 (PMC13638210; doi:10.1099/jmm.0.002209)
Supplement: Supplementary file 1 [file jmm-75-02209-s001.pdf]

Supplementary Data S1. DDDkr values for all antimicrobial agents used in cattle

Antimicrobial	Administration route	Product type	No of product	Mean = DDDkr[a]	Median	Mode	Min	Max	% products <90% from consensus DDDkr	% products > 110% from consensus DDDkr	Difference highest vs lowest (%)	Day[b]	DCDkr[c]	DDDvet[d] (mg/kg)	QDDD[e]	Mean/ Median Ratio
**Amoxicillin**	**Oral**	Single	48	7.1	7.5	6.5	6.5	9.8	0.0	1.5	150.0	4.4	31.2	20.0	0.35	0.9
**Amoxicillin**	**Oral**	Combination	3	5.7	3.3	3.3	3.3	10.5	50.0	25.0	323.1	4.0	22.7	-	-	1.7
**Amoxicillin**	**Injection**	Single	15	13.1	15.0	15.0	7.0	15.0	31.3	62.5	214.3	1.8	23.6	8.3	1.58	0.9
**Ampicillin**	**Oral**	Single	44	9.5	9.5	9.5	9.5	9.5	0.0	0.0	100.0	5.0	47.3	29.0	0.33	1.0
**Ampicillin**	**Oral**	Combination	14	5.0	5.0	5.0	5.0	5.0	0.0	0.0	100.0	5.0	25.0	-	-	1.0
**Ampicillin**	**Injection**	Single	11	7.8	7.5	7.5	7.5	11.3	0.0	9.1	150.0	1.3	10.3	11.0	0.71	1.0
**Ampicillin**	**Injection**	Combination	1	10.0	10.0	-	10.0	10.0	0.0	0.0	100.0	1.0	10.0	-	-	1.0
**Ampicillin**	**Intramammary**	Combination	1	0.4	0.4	-	0.4	0.4	0.0	0.0	100.0	4.0	1.6	-	-	1.0
**Apramycin**	**Oral**	Single	1	30.0	30.0	-	30.0	30.0	0.0	0.0	100.0	5.0	150.0	30.0	1.00	1.0
**Apramycin**	**Injection**	Single	4	20.0	20.0	20.0	20.0	20.0	0.0	0.0	100.0	5.0	100.0	20.0	1.00	1.0
**Cefquinome**	**Injection**	Single	6	1.0	1.0	1.0	1.0	1.0	0.0	0.0	100.0	4.0	4.0	1.5	0.67	1.0
**Cefquinome**	**Intramammary**	Single	1	0.9	0.9	-	0.9	0.9	0.0	0.0	100.0	1.5	1.4	-	-	1.0
**Ceftiofur**	**Injection**	Single	40	1.0	1.0	1.0	1.0	1.0	0.0	0.0	100.0	3.0	3.0	1.0	1.00	1.0
**Ceftiofur**	**Intramammary**	Combination	1	0.6	0.6	-	0.6	0.6	0.0	0.0	100.0	4.0	2.5	-	-	1.0
**Cefuroxime**	**Intramammary**	Single	2	3.1	3.1	3.1	3.1	3.1	0.0	0.0	100.0	1.5	4.7	-	-	1.0
**Cephalexin**	**Intramammary**	Combination	6	0.7	0.4	0.2	0.2	2.0	66.7	33.3	1280.0	3.8	2.6	-	-	1.6
**Cephalonium**	**Intramammary**	Single	3	0.4	0.4	0.4	0.4	0.4	0.0	0.0	100.0	4.0	1.6	-	-	1.0
**Cephapirin**	**Intrauterine**	Single	1	0.2	0.2	-	0.2	0.2	0.0	0.0	100.0	4.0	0.8	-	-	1.0
**Cephazolin**	**Injection**	Single	1	2.5	2.5	-	2.5	2.5	0.0	0.0	100.0	4.0	10.0	-	-	1.0
**Cloxacillin**	**Intramammary**	Combination	2	0.8	0.8	0.8	0.8	0.8	0.0	0.0	100.0	4.0	3.1	-	-	1.0
**Colistin**	**Oral**	Single	27	0.5	0.5	0.5	0.5	0.5	0.0	0.0	100.0	1.0	0.5	4.8	0.09	1.0
**Colistin**	**Oral**	Combination	17	0.3	0.2	0.2	0.2	0.6	56.5	13.0	262.5	4.8	1.5	-	-	1.2
**Colistin**	**Injection**	Combination	3	1.4	1.2	1.2	1.2	1.8	66.7	33.3	150.0	1.7	2.4	2.6	0.55	1.2
**Dicloxacillin**	**Intramammary**	Single	2	0.8	0.8	0.8	0.8	0.8	0.0	0.0	100.0	4.0	3.1	-	-	1.0
**Dihydrostreptomycin**	**Injection**	Combination	14	5.7	5.0	5.0	3.0	10.0	66.7	20.0	333.3	3.1	17.8	25.0	0.23	1.1
**Dihydrostreptomycin**	**Intramammary**	Combination	1	1.9	1.9	-	1.9	1.9	0.0	0.0	100.0	3.0	5.7	-	-	1.0

**Enrofloxacin**	**Oral**	Single	1	3.8	3.8	-	3.8	3.8	0.0	0.0	100.0	4.0	15.0	4.7	0.80	1.0
**Enrofloxacin**	**Injection**	Single	31	4.5	4.2	4.2	3.8	7.5	2.9	8.8	200.0	3.7	16.5	4.2	1.07	1.1
**Enrofloxacin**	**Intramammary**	Combination	1	2.5	2.5	-	2.5	2.5	0.0	0.0	100.0	3.0	7.6	-	-	1.0
**Erythromycin**	**Oral**	Combination	2	27.0	27.0	-	27.0	27.0	0.0	0.0	100.0	4.0	108.0	20.0	1.35	1.0
**Florfenicol**	**Spray**	Combination	2	0.4	0.4	0.4	0.4	0.4	0.0	0.0	100.0	1.0	0.4	-	-	1.0
**Florfenicol**	**Injection**	Single	16	18.8	20.0	20.0	10.0	20.0	12.5	0.0	200.0	2.3	42.2	13.0	1.44	0.9
**Gentamycin**	**Injection**	Single	8	10.0	10.0	10.0	10.0	10.0	0.0	0.0	100.0	1.0	10.0	8.4	1.19	1.0
**Gentamycin**	**Intramammary**	Single	1	0.9	0.9	-	0.9	0.9	0.0	0.0	100.0	3.0	2.6	-	-	1.0
**Gentamycin**	**Intramammary**	Combination	1	0.7	0.7	-	0.7	0.7	0.0	0.0	100.0	4.0	2.6	-	-	1.0
**Lincomycin**	**Spray**	Combination	2	0.1	0.1	0.1	0.1	0.1	0.0	0.0	100.0	1.0	0.1	-	-	1.0
**Lincomycin**	**Injection**	Combination	4	5.4	5.6	5.6	5.0	5.6	0.0	0.0	111.0	3.0	16.2	13.0	0.42	1.0
**Marbofloxacin**	**Injection**	Single	18	2.4	2.0	2.0	2.0	10.0	81.0	4.8	500.0	3.8	9.2	3.6	0.68	1.2
**Minocycline**	**Oral**	Combination	1	6.7	6.7	-	6.7	6.7	0.0	0.0	100.0	1.0	6.7	-	-	1.0
**Neomycin**	**Oral**	Single	2	8.9	8.9	-	7.7	10.0	6.7	6.7	129.9	2.0	17.7	21.0	0.42	1.0
**Neomycin**	**Oral**	Combination	3	2.4	2.6	2.6	2.0	2.6	20.0	0.0	130.1	3.5	8.4	-	-	0.9
**Neomycin**	**Injection**	Combination	1	7.5	7.5	-	7.5	7.5	0.0	0.0	100.0	1.0	7.5	7.4	1.01	1.0
**Neomycin**	**Intramammary**	Combination	8	0.8	0.6	0.6	0.4	1.9	75.0	12.5	480.0	2.8	2.1	-	-	1.2
**Novobiocin**	**Intramammary**	Combination	1	1.3	1.3	-	1.3	1.3	0.0	0.0	100.0	3.0	3.8	-	-	1.0
**Oxytetracycline**	**Oral**	Single	9	22.0	22.0	22.0	21.6	22.2	0.0	0.0	102.8	10.5	230.8	20.0	1.10	1.0
**Oxytetracycline**	**Oral**	Combination	2	26.0	26.0	26.0	26.0	26.0	0.0	0.0	100.0	4.0	104.0	-	-	1.0
**Oxytetracycline**	**Injection**	Single	5	15.5	20.0	20.0	8.8	20.0	40.0	60.0	227.3	1.0	15.5	6.5	2.39	0.8
**Penicillin**	**Injection**	Single	4	5.2	4.0	3.6	3.6	9.0	75.0	25.0	250.0	2.3	11.6	14.0	0.37	1.3
**Penicillin**	**Injection**	Combination	19	5.8	5.6	8.0	1.8	13.5	35.0	35.0	750.1	2.9	16.7	2.9	1.99	1.0
**Penicillin**	**Intramammary**	Combination	1	0.4	0.4	-	0.4	0.4	0.0	0.0	100.0	1.0	0.4	-	-	1.0
**Phthalylsulfathiazole**	**Oral**	Combination	1	30.0	30.0	-	30.0	30.0	0.0	0.0	100.0	2.5	75.0	25.0	1.20	1.0
**Rifaximin**	**Intrauterine**	Single	1	0.5	0.5	-	0.5	0.5	0.0	0.0	100.0	4.0	1.9	-	-	1.0
**Rifaximin**	**Intramammary**	Single	1	0.2	0.2	-	0.2	0.2	0.0	0.0	100.0	4.0	0.6	-	-	1.0
**Spectinomycin**	**Injection**	Combination	5	11.8	11.1	11.1	11.1	12.9	0.0	0.0	115.6	3.2	37.8	28.0	0.42	1.1
**Spiramycin**	**Injection**	Combination	2	26.5	26.5	-	3.0	50.0	50.0	50.0	1666.7	3.0	79.5	21.0	1.26	1.0
**Streptomycin**	**Oral**	Combination	1	35.8	35.8	-	35.8	35.8	0.0	0.0	100.0	2.5	89.5	70.0	0.51	1.0

**Streptomycin**	**Intramammary**	Combination	1	0.6	0.6	-	0.6	0.6	0.0	0.0	100.0	1.0	0.6	-	-	1.0
**Sulfadiazine**	**Oral**	Combination	2	22.5	22.5	22.5	22.5	22.5	0.0	0.0	100.0	4.0	90.0	24.0	0.94	1.0
**Sulfadiazine**	**Injection**	Combination	2	8.7	8.7	-	4.8	12.5	50.0	50.0	260.4	4.0	34.6	13.0	0.67	1.0
**Sulfadimethoxine**	**Injection**	Single	1	40.0	40.0	-	40.0	40.0	0.0	0.0	100.0	5.0	200.0	30.0	1.33	1.0
**Sulfadimethoxine**	**Injection**	Combination	1	4.3	4.3	-	4.3	4.3	0.0	0.0	100.0	3.0	13.0	19.0	0.23	1.0
**Sulfadoxine**	**Injection**	Combination	3	12.6	12.5	-	12.0	13.3	0.0	0.0	111.1	2.3	29.4	14.0	0.90	1.0
**Sulfaguanidine**	**Oral**	Combination	1	50.0	50.0	-	50.0	50.0	0.0	0.0	100.0	2.5	125.0	77.0	0.65	1.0
**Sulfamerazine**	**Injection**	Combination	1	4.8	4.8	-	4.8	4.8	0.0	0.0	100.0	7.0	33.6	-	-	1.0
**Sulfamethazine**	**Injection**	Combination	1	13.3	13.3	-	13.3	13.3	0.0	0.0	100.0	3.0	40.0	-	-	1.0
**Sulfamethoxazole**	**Oral**	Combination	3	16.1	12.5	-	9.0	26.7	6.9	3.4	296.3	3.7	58.9	-	-	1.3
**Sulfamethoxazole**	**Injection**	Combination	1	6.5	6.5	-	6.5	6.5	0.0	0.0	100.0	3.0	19.5	15.0	0.43	1.0
**Sulfamethoypyridazine**	**Oral**	Combination	1	20.0	20.0	-	20.0	20.0	0.0	0.0	100.0	4.0	80.0	23.0	0.87	1.0
**Sulfathiazole**	**Oral**	Combination	1	30.0	30.0	-	30.0	30.0	0.0	0.0	100.0	2.5	75.0	-	-	1.0
**Sulfathiazole**	**Injection**	Combination	1	3.2	3.2	-	3.2	3.2	0.0	0.0	100.0	7.0	22.4	-	-	1.0
**Sulfathiazole**	**Intramammary**	Single	1	3.1	3.1	-	3.1	3.1	0.0	0.0	100.0	5.0	15.7	-	-	1.0
**Thiamphenicol**	**Injection**	Combination	1	9.0	9.0	-	9.0	9.0	0.0	0.0	100.0	1.0	9.0	-	-	1.0
**Tildipirosin**	**Injection**	Single	2	4.0	4.0	4.0	4.0	4.0	0.0	0.0	100.0	1.0	4.0	-	-	1.0
**Tilmicosin**	**Injection**	Single	6	10.0	10.0	10.0	10.0	10.0	0.0	0.0	100.0	1.0	10.0	4.0	2.50	1.0
**Trimethoprim**	**Oral**	Combination	4	4.6	4.5	4.5	4.0	5.3	2.5	2.5	133.3	3.3	14.9	4.8	0.95	1.0
**Trimethoprim**	**Injection**	Combination	7	2.7	2.7	2.5	2.4	3.2	0.0	12.5	133.3	3.0	8.0	2.8	0.95	1.0
**Tulathromycin**	**Injection**	Single	21	2.5	2.5	2.5	2.5	2.5	0.0	0.0	100.0	1.0	2.5	-	-	1.0
**Tylosin**	**Oral**	Single	18	0.2	0.2	0.2	0.2	0.2	0.0	0.0	100.0	21.0	4.2	41.0	0.005	1.0
**Tylosin**	**Injection**	Single	5	8.6	7.0	7.0	7.0	15.0	80.0	20.0	214.3	2.6	22.4	13.0	0.66	1.2
**Tylosin**	**Injection**	Combination	3	4.9	5.0	-	2.6	7.0	10.0	10.0	270.5	2.3	11.3	-	-	1.0

[a] DDDkr defined daily doses of the active substance used in Korea.

[b] day mean treatment duration (days) from product labels.

[c] DCDkr defined course doses of the active substance used in Korea.

[d] DDDvet defined daily doses of the active substance used in Europe established by the European Medicines Agency.

[e] QDDD DDDkr/DDDvet.

Supplementary Data S2. DDDkr values for all antimicrobial agents used in pigs

Antimicrobial	Administration route	Product type	No of product	Mean = DDDkr[a]	Median	Mode	Min	Max	% products <90% from consensus DDDkr	% products > 110% from consensus DDDkr	Difference highest vs lowest (%)	Day[b]	DCDkr[c]	DDDvet[d] (mg/kg)	QDDD[e]	Mean/ Median Ratio
**Amoxicillin**	**Oral**	Single	64	7.6	7.5	7.5	6.5	20.0	35.4	7.7	307.7	4.5	34.6	17.0	0.450	1.0
**Amoxicillin**	**Oral**	Combination	4	5.1	3.3	3.3	3.3	10.5	75.0	25.0	323.1	4.0	20.3	-	-	1.6
**Amoxicillin**	**Injection**	Single	16	12.9	15.0	15.0	7.0	15.0	37.5	62.5	214.3	1.9	24.3	8.9	1.454	0.9
**Ampicillin**	**Oral**	Single	44	12.5	12.5	12.5	12.5	12.5	0.0	0.0	100.0	5.0	62.2	30.0	0.417	1.0
**Ampicillin**	**Oral**	Combination	14	5.0	5.0	5.0	5.0	5.0	0.0	0.0	100.0	5.0	25.0	-	-	1.0
**Ampicillin**	**Injection**	Single	11	7.8	7.5	7.5	7.5	11.3	0.0	9.1	150.0	1.3	10.3	12.0	0.653	1.0
**Ampicillin**	**Injection**	Combination	1	10.0	10.0	-	10.0	10.0	0.0	0.0	100.0	1.0	10.0	-	-	1.0
**Apramycin**	**Oral**	Single	4	5.0	4.0	4.0	2.0	10.0	60.0	20.0	500.0	16.5	82.5	9.0	0.556	1.3
**Apramycin**	**Injection**	Single	3	20.0	20.0	20.0	20.0	20.0	0.0	0.0	100.0	3.0	60.0	-	-	1.0
**Bacitracin**	**Oral**	Single	17	7.2	4.0	4.0	4.0	14.0	55.0	30.0	350.0	2.5	18.2	-	-	1.8
**Cefquinome**	**Injection**	Single	6	2.0	2.0	2.0	2.0	2.0	0.0	0.0	100.0	3.0	6.0	1.9	1.053	1.0
**Ceftiofur**	**Injection**	Single	48	4.2	4.0	4.0	4.0	5.0	0.0	16.7	125.0	2.7	11.1	3.0	1.389	1.0
**Chlortetracycline**	**Oral**	Single	13	11.4	8.8	8.8	6.0	22.0	71.4	21.4	366.7	10.2	115.9	31.0	0.368	1.3
**Chlortetracycline**	**Oral**	Combination	24	9.2	9.9	9.9	4.0	10.0	16.7	0.0	250.0	6.8	62.4	-	-	0.9
**Colistin**	**Oral**	Single	27	1.2	1.2	1.2	1.2	1.2	0.0	0.0	100.0	1.0	1.2	5.0	0.240	1.0
**Colistin**	**Oral**	Combination	22	0.8	0.5	0.5	0.3	2.0	78.3	17.4	600.4	4.8	3.6	-	-	1.5
**Colistin**	**Injection**	Combination	2	1.2	1.2	1.2	1.2	1.2	0.0	0.0	100.0	2.0	2.4	2.7	0.452	1.0
**Dihydrostreptomycin**	**Injection**	Combination	15	8.9	6.0	5.0	5.0	25.0	66.7	33.3	500.0	3.0	26.7	20.0	0.444	1.5
**Doxycycline**	**Oral**	Single	4	10.0	10.0	10.0	10.0	10.0	0.0	0.0	100.0	5.0	50.0	11.0	0.909	1.0
**Enramycin**	**Oral**	Single	3	1.2	1.2	1.2	1.2	1.2	0.0	0.0	100.0	8.0	9.6	-	-	1.0
**Enrofloxacin**	**Oral**	Single	8	3.6	4.0	4.0	1.0	4.0	3.6	25.0	400.0	5.6	20.2	2.5	1.450	0.9
**Enrofloxacin**	**Oral**	Combination	1	1.6	1.6	-	1.6	1.6	0.0	0.0	100.0	5.0	8.0	-	-	1.0
**Enrofloxacin**	**Injection**	Single	34	4.6	4.2	4.2	3.8	7.5	2.9	11.8	200.0	3.7	16.7	3.4	1.344	1.1
**Florfenicol**	**Oral**	Single	107	5.8	10.0	10.0	1.2	10.0	46.8	51.4	833.3	6.0	34.7	10.0	0.582	0.6
**Florfenicol**	**Spray**	Combination	2	2.0	2.0	2.0	2.0	2.0	0.0	0.0	100.0	1.0	2.0	-	-	1.0
**Florfenicol**	**Injection**	Single	16	14.1	15.0	15.0	7.5	15.0	12.5	0.0	200.0	2.3	31.6	-	-	0.9
**Florfenicol**	**Injection**	Combination	5	8.0	10.0	10.0	5.0	10.0	33.3	50.0	200.0	2.4	19.2	9.5	0.842	0.8

**Flumequine**	**Oral**	Single	2	20.0	20.0	20.0	20.0	20.0	0.0	0.0	100.0	1.0	20.0	16.0	1.250	1.0
**Gentamycin**	**Oral**	Single	3	1.1	1.1	1.1	1.1	1.1	0.0	0.0	100.0	3.0	3.3	1.4	0.786	1.0
**Gentamycin**	**Injection**	Single	9	9.4	10.0	10.0	5.0	10.0	11.1	0.0	200.0	1.0	9.4	6.1	1.548	0.9
**Kanamycin**	**Spray**	Single	7	4.4	4.4	4.4	4.4	4.4	0.0	0.0	100.0	31.5	138.8	-	-	1.0
**Kanamycin**	**Injection**	Single	1	10.0	10.0	-	10.0	10.0	0.0	0.0	100.0	1.0	10.0	28.0	0.357	1.0
**Kitasamycin**	**Oral**	Combination	1	8.8	8.8	-	8.8	8.8	0.0	0.0	100.0	6.0	52.8	-	-	1.0
**Lincomycin**	**Oral**	Single	8	7.0	7.0	7.0	7.0	7.0	0.0	0.0	100.0	21.0	147.8	7.6	0.926	1.0
**Lincomycin**	**Oral**	Combination	24	3.4	3.5	3.5	2.0	5.0	32.0	16.0	250.3	17.1	58.6	2.2	1.554	1.0
**Lincomycin**	**Spray**	Combination	2	0.4	0.4	0.4	0.4	0.4	0.0	0.0	100.0	1.0	0.4	-	-	1.0
**Lincomycin**	**Injection**	Single	1	15.0	15.0	-	15.0	15.0	0.0	0.0	100.0	5.0	75.0	10.0	1.500	1.0
**Lincomycin**	**Injection**	Combination	5	5.3	5.6	5.6	5.0	5.6	0.0	0.0	111.0	2.9	15.5	7.8	0.683	1.0
**Marbofloxacin**	**Injection**	Single	20	2.3	2.0	2.0	2.0	8.0	90.5	4.8	400.0	3.8	8.6	2.0	1.150	1.2
**Marbofloxacin**	**Injection**	Combination	1	2.0	2.0	-	2.0	2.0	0.0	0.0	100.0	3.0	6.0	-	-	1.0
**Minocycline**	**Oral**	Combination	1	6.7	6.7	-	6.7	6.7	0.0	0.0	100.0	1.0	6.7	-	-	1.0
**Neomycin**	**Oral**	Single	14	16.2	19.0	19.0	3.1	19.0	20.0	73.3	620.0	4.3	69.6	25.0	0.650	0.9
**Neomycin**	**Oral**	Combination	3	3.1	3.8	3.8	1.7	3.8	20.0	40.0	225.1	5.5	16.8	-	-	0.8
**Oxytetracycline**	**Oral**	Single	9	22.0	22.0	22.0	21.6	22.2	0.0	0.0	102.8	10.5	230.8	26.0	0.845	1.0
**Oxytetracycline**	**Oral**	Combination	2	10.1	10.1	-	9.6	10.6	0.0	0.0	110.0	8.5	85.7	-	-	1.0
**Oxytetracycline**	**Injection**	Single	5	15.5	20.0	20.0	8.8	20.0	40.0	60.0	227.3	1.0	15.5	7.5	2.069	0.8
**Penicillin**	**Oral**	Combination	6	5.1	4.5	4.5	2.0	13.0	83.3	16.7	648.0	9.8	50.2	10.0	0.511	1.1
**Penicillin**	**Injection**	Single	4	5.2	4.0	3.6	3.6	9.0	75.0	25.0	250.0	2.3	11.6	13.0	0.398	1.3
**Penicillin**	**Injection**	Combination	20	7.8	5.6	8.0	2.9	22.5	60.0	15.0	781.4	2.8	21.9	-	-	1.4
**Phthalylsulfathiazole**	**Oral**	Combination	1	25.0	25.0	-	25.0	25.0	0.0	0.0	100.0	2.5	62.5	-	-	1.0
**Spectinomycin**	**Oral**	Single	1	22.2	22.2	-	22.2	22.2	0.0	0.0	100.0	4.0	88.9	33.0	0.673	1.0
**Spectinomycin**	**Oral**	Combination	19	2.8	2.6	2.6	2.6	4.0	0.0	10.5	151.7	19.5	54.4	3.4	0.819	1.1
**Spectinomycin**	**Injection**	Combination	6	10.6	10.6	11.1	10.0	11.1	0.0	0.0	111.3	3.0	31.7	17.0	0.621	1.0
**Spiramycin**	**Oral**	Single	2	8.0	8.0	8.0	8.0	8.0	0.0	0.0	100.0	10.0	80.0	25.0	0.320	1.0
**Spiramycin**	**Injection**	Combination	2	30.5	30.5	-	11.0	50.0	50.0	50.0	454.5	3.0	91.5	24.0	1.271	1.0
**Streptomycin**	**Oral**	Combination	5	21.4	15.0	15.0	15.0	32.4	60.0	40.0	216.0	6.7	143.7	-	-	1.4
**Sulfachloropyridazine**	**Oral**	Combination	6	20.0	20.0	20.0	20.0	20.0	0.0	0.0	100.0	7.5	150.0	20.0	1.000	1.0

**Sulfadiazine**	**Oral**	Combination	1	22.5	22.5	-	22.5	22.5	0.0	0.0	100.0	5.0	112.5	23.0	0.978	1.0
**Sulfadimethoxine**	**Injection**	Single	1	40.0	40.0	-	40.0	40.0	0.0	0.0	100.0	5.0	200.0	30.0	1.333	1.0
**Sulfadimethoxine**	**Injection**	Combination	1	4.3	4.3	-	4.3	4.3	0.0	0.0	100.0	3.0	13.0	19.0	0.228	1.0
**Sulfadoxine**	**Injection**	Combination	3	12.6	12.5	-	12.0	13.3	0.0	0.0	111.1	2.3	29.4	14.0	0.901	1.0
**Sulfaguanidine**	**Oral**	Combination	1	41.7	41.7	-	41.7	41.7	0.0	0.0	100.0	2.5	104.2	54.0	0.772	1.0
**Sulfamethazine**	**Oral**	Combination	18	4.9	4.4	4.4	4.4	8.8	83.3	16.7	200.0	18.4	90.7	-	-	1.1
**Sulfamethoxazole**	**Oral**	Combination	29	14.7	18.0	18.0	4.0	26.7	37.9	62.1	666.7	4.6	68.0	20.0	0.736	0.8
**Sulfamethoxazole**	**Injection**	Combination	2	9.9	9.9	-	6.5	13.3	50.0	50.0	205.1	3.0	29.8	20.0	0.496	1.0
**Sulfathiazole**	**Oral**	Combination	3	11.1	4.4	-	4.0	25.0	66.7	33.3	625.3	10.2	113.2	-	-	2.5
**Thiamphenicol**	**Injection**	Combination	1	20.0	20.0	-	20.0	20.0	0.0	0.0	100.0	1.0	20.0	75.0	0.267	1.0
**Tiamulin**	**Oral**	Single	49	7.7	6.2	11.9	3.2	12.2	57.1	34.7	380.9	8.0	62.1	9.7	0.798	1.2
**Tiamulin**	**Oral**	Combination	24	3.2	3.3	3.3	0.9	4.0	4.2	8.3	454.5	6.1	19.9	-	-	1.0
**Tiamulin**	**Injection**	Single	2	12.5	12.5	12.5	12.5	12.5	0.0	0.0	100.0	3.3	40.6	12.0	1.042	1.0
**Tilmicosin**	**Oral**	Single	54	16.9	20.0	20.0	10.0	20.0	37.0	55.6	200.0	10.9	184.1	15.0	1.123	0.8
**Trimethoprim**	**Oral**	Combination	36	3.2	3.6	3.6	0.8	5.3	25.0	65.0	666.7	5.1	16.4	4.7	0.683	0.9
**Trimethoprim**	**Injection**	Combination	4	2.6	2.6	2.7	2.4	2.7	0.0	0.0	111.1	2.5	6.4	3.0	0.853	1.0
**Tulathromycin**	**Injection**	Single	21	2.5	2.5	2.5	2.5	2.5	0.0	0.0	100.0	1.0	2.5	-	-	1.0
**Tylosin**	**Oral**	Single	32	10.8	4.4	4.4	4.4	25.0	66.7	30.3	568.2	16.0	172.9	12.0	0.903	2.5
**Tylosin**	**Oral**	Combination	13	4.4	4.4	4.4	4.4	4.4	0.0	0.0	100.0	21.0	92.4	-	-	1.0
**Tylosin**	**Injection**	Single	5	6.2	6.0	6.0	6.0	7.0	0.0	20.0	116.7	2.6	16.1	13.0	0.477	1.0
**Tylosin**	**Injection**	Combination	10	3.4	2.5	2.5	2.2	6.0	70.0	30.0	272.7	2.4	8.1	-	-	1.4
**Tylvalosin**	**Oral**	Single	2	2.1	2.1	-	1.4	2.8	50.0	50.0	200.0	7.0	14.7	3.6	0.583	1.0
**Virginiamycin**	**Oral**	Single	1	0.6	0.6	-	0.6	0.6	50.0	50.0	100.0	1.0	0.6	-	-	1.0

[a] DDDkr defined daily doses of the active substance used in Korea.

[b] day mean treatment duration (days) from product labels.

[c] DCDkr defined course doses of the active substance used in Korea.

[d] DDDvet defined daily doses of the active substance used in Europe established by the European Medicines Agency.

[e] QDDD DDDkr/DDDvet.

Supplementary Data S3. DDDkr values for all antimicrobial agents used in pourtry

Antimicrobial	Administration route	Product type	No of product	Mean = DDDkr[a]	Median	Mode	Min	Max	% products <90% from consensus DDDkr	% products > 110% from consensus DDDkr	Difference highest vs lowest (%)	Day[b]	DCDkr[c]	DDDvet[d] (mg/kg)	QDDD[e]	Mean/ Median Ratio
**Amoxicillin**	**Oral**	Single	26	30.0	30.0	30.0	15.0	45.0	1.5	1.5	300.0	4.7	141.9	16.00	1.875	1.0
**Amoxicillin**	**Oral**	Combination	1	33.0	33.0	-	33.0	33.0	0.0	0.0	100.0	4.0	132.0	-	-	1.0
**Ampicillin**	**Oral**	Single	44	15.1	15.0	15.0	15.0	20.0	0.0	2.3	133.3	5.0	75.2	108.00	0.140	1.0
**Ampicillin**	**Oral**	Combination	14	11.5	11.5	11.5	11.5	11.5	0.0	0.0	100.0	5.0	57.5	-	-	1.0
**Apramycin**	**Oral**	Single	2	43.1	43.1	43.1	43.1	43.1	0.0	0.0	100.0	5.0	215.6	81.00	0.532	1.0
**Bacitracin**	**Oral**	Single	20	8.6	6.3	3.9	3.9	21.5	55.0	20.0	550.0	3.0	25.7	-	-	1.4
**Chlortetracycline**	**Oral**	Single	14	32.0	33.6	42.9	13.2	50.1	50.0	50.0	379.2	6.5	208.0	30.00	1.066	1.0
**Chlortetracycline**	**Oral**	Combination	10	25.9	25.9	26.0	25.7	26.0	0.0	0.0	101.0	5.2	134.5	-	-	1.0
**Colistin**	**Oral**	Single	17	1.1	0.7	0.7	0.7	2.4	41.9	12.9	375.2	1.9	2.1	5.10	0.210	1.6
**Colistin**	**Oral**	Combination	20	3.1	1.6	1.6	1.4	9.0	65.2	21.7	640.0	4.9	15.2	-	-	2.0
**Doxycycline**	**Oral**	Single	5	13.8	11.5	11.5	11.5	17.3	50.0	33.3	150.0	4.6	63.5	15.00	0.920	1.2
**Enramycin**	**Oral**	Single	3	1.1	1.1	1.1	1.1	1.1	0.0	0.0	100.0	8.0	8.8	-	-	1.0
**Enrofloxacin**	**Oral**	Single	19	11.5	11.5	11.5	11.5	11.5	0.0	0.0	100.0	3.9	45.4	10.00	1.150	1.0
**Erythromycin**	**Oral**	Combination	2	20.7	20.7	20.7	20.7	20.7	0.0	0.0	100.0	5.0	103.5	20.00	1.035	1.0
**Florfenicol**	**Oral**	Single	56	20.0	20.0	20.0	20.0	20.0	0.0	0.0	100.0	5.0	100.7	30.00	0.667	1.0
**Flumequine**	**Oral**	Single	4	12.0	12.0	12.0	12.0	12.0	0.0	0.0	100.0	4.0	48.0	14.00	0.857	1.0
**Gentamycin**	**Injection**	Single	8	2.0	2.0	2.0	2.0	2.0	0.0	0.0	100.0	1.0	2.0	-	-	1.0
**Lincomycin**	**Oral**	Single	9	0.4	0.4	0.4	0.4	0.4	0.0	0.0	100.0	1.0	0.4	8.60	0.050	1.0
**Lincomycin**	**Oral**	Combination	23	15.2	11.4	11.4	8.6	38.3	64.0	24.0	446.3	15.9	241.3	22.00	0.691	1.3
**Lincomycin**	**Injection**	Combination	3	21.7	10.0	10.0	10.0	45.0	40.0	20.0	450.5	3.0	65.0	-	-	2.2
**Neomycin**	**Oral**	Single	11	12.0	12.0	12.0	12.0	12.0	0.0	0.0	100.0	3.0	36.0	24.00	0.500	1.0
**Penicillin**	**Oral**	Combination	1	6.2	6.2	-	6.2	6.2	0.0	0.0	100.0	10.0	62.4	-	-	1.0
**Roxithromycine**	**Oral**	Combination	1	2.3	2.3	-	2.3	2.3	0.0	0.0	100.0	5.0	11.5	-	-	1.0
**Spectinomycin**	**Oral**	Combination	17	16.6	8.6	8.6	8.6	76.8	78.9	10.5	894.7	18.9	313.5	38.00	0.437	1.9
**Spectinomycin**	**Injection**	Combination	3	20.0	20.0	20.0	20.0	20.0	0.0	0.0	100.0	3.0	60.1	-	-	1.0
**Streptomycin**	**Oral**	Combination	1	15.6	15.6	-	15.6	15.6	0.0	0.0	100.0	10.0	156.0	-	-	1.0
**Sulfachloropyridazine**	**Oral**	Combination	6	20.0	20.0	20.0	20.0	20.0	0.0	0.0	100.0	4.5	90.0	30.00	0.667	1.0

**Sulfadiazine**	**Oral**	Combination	2	17.3	17.3	17.3	17.3	17.3	0.0	0.0	100.0	5.0	86.3	34.00	0.507	1.0
**Sulfamethoxazole**	**Oral**	Combination	16	28.5	26.0	26.0	15.5	46.0	3.4	3.4	296.3	5.1	146.0	27.00	1.055	1.1
**Sulfaquinoxaline**	**Oral**	Combination	3	26.8	23.0	-	23.0	34.5	66.7	33.3	150.0	5.7	152.1	60.00	0.447	1.2
**Tiamulin**	**Oral**	Single	26	38.8	43.1	43.1	10.4	43.1	6.1	34.7	414.9	4.2	161.2	23.00	1.688	0.9
**Tiamulin**	**Oral**	Combination	9	9.3	8.6	8.6	8.6	15.2	0.0	4.2	176.9	5.6	51.7	-	-	1.1
**Tiamulin**	**Injection**	Single	1	2.5	2.5	-	2.5	2.5	0.0	0.0	100.0	1.0	2.5	-	-	1.0
**Tilmicosin**	**Oral**	Single	34	17.0	17.3	17.3	15.5	17.3	0.0	0.0	111.1	4.0	68.2	18.00	0.947	1.0
**Trimethoprim**	**Oral**	Combination	18	5.2	5.2	5.2	3.5	11.5	20.0	5.0	334.0	5.2	27.0	6.40	0.809	1.0
**Tylosin**	**Oral**	Single	11	115	115	115	115	115	0.0	0.0	100.0	4.8	554.1	81.00	1.420	1.0
**Tylosin**	**Oral**	Combination	1	2.3	2.3	-	2.3	2.3	0.0	0.0	100.0	5.0	11.5	-	-	1.0
**Tylosin**	**Injection**	Single	1	15.0	15.0	-	15.0	15.0	0.0	0.0	100.0	1.0	15.0	-	-	1.0
**Tylvalosin**	**Oral**	Single	1	45.5	45.5	-	45.5	45.5	0.0	0.0	100.0	4.0	182.0	-	-	1.0
**Virginiamycin**	**Oral**	Single	1	1.3	1.3	-	1.3	1.3	0.0	0.0	100.0	1.0	1.3	-	-	1.0

[a] DDDkr defined daily doses of the active substance used in Korea.

[b] day mean treatment duration (days) from product labels.

[c] DCDkr defined course doses of the active substance used in Korea.

[d] DDDvet defined daily doses of the active substance used in Europe established by the European Medicines Agency.

[e] QDDD DDDkr/DDDvet.
